# Simultaneous Highly Efficient Contrast‐Free Lumen and Vessel Wall MR Imaging for Anatomical Assessment of Aortic Disease

**DOI:** 10.1002/jmri.28613

**Published:** 2023-02-09

**Authors:** Camila Munoz, Anastasia Fotaki, Alina Hua, Reza Hajhosseiny, Karl P. Kunze, Tevfik F. Ismail, Radhouene Neji, Kuberan Pushparajah, René M. Botnar, Claudia Prieto

**Affiliations:** ^1^ School of Biomedical Engineering and Imaging Sciences King's College London London UK; ^2^ MR Research Collaborations Siemens Healthcare Limited Frimley UK; ^3^ Escuela de Ingeniería, Pontificia Universidad Católica de Chile Santiago Chile; ^4^ Instituto de Ingeniería Biológica y Médica, Pontificia Universidad Católica de Chile Santiago Chile; ^5^ Millenium Institute for Intelligent Healthcare Engineering iHEALTH Santiago Chile

**Keywords:** aortic MR angiography, multicontrast imaging, vessel wall imaging

## Abstract

**Background:**

Bright‐blood lumen and black‐blood vessel wall imaging are required for the comprehensive assessment of aortic disease. These images are usually acquired separately, resulting in long examinations and potential misregistration between images.

**Purpose:**

To characterize the performance of an accelerated and respiratory motion‐compensated three‐dimensional (3D) cardiac MRI technique for simultaneous contrast‐free aortic lumen and vessel wall imaging with an interleaved T2 and inversion recovery prepared sequence (iT2Prep‐BOOST).

**Study Type:**

Prospective.

**Population:**

A total of 30 consecutive patients with aortopathy referred for a clinically indicated cardiac MRI examination (9 females, mean age ± standard deviation: 32 ± 12 years).

**Field Strength/Sequence:**

1.5‐T; bright‐blood MR angiography (diaphragmatic navigator‐gated T2‐prepared 3D balanced steady‐state free precession [bSSFP], T2Prep‐bSSFP), breath‐held black‐blood two‐dimensional (2D) half acquisition single‐shot turbo spin echo (HASTE), and 3D bSSFP iT2Prep‐BOOST.

**Assessment:**

iT2Prep‐BOOST bright‐blood images were compared to T2prep‐bSSFP images in terms of aortic vessel dimensions, lumen‐to‐myocardium contrast ratio (CR), and image quality (diagnostic confidence, vessel sharpness and presence of artifacts, assessed by three cardiologists on a 4‐point scale, 1: nondiagnostic to 4: excellent). The iT2Prep‐BOOST black‐blood images were compared to 2D HASTE images for quantification of wall thickness. A visual comparison between computed tomography (CT) and iT2Prep‐BOOST was performed in a patient with chronic aortic dissection.

**Statistical Tests:**

Paired *t*‐tests, Wilcoxon signed‐rank tests, intraclass correlation coefficient (ICC), Bland–Altman analysis. A *P* value < 0.05 was considered statistically significant.

**Results:**

Bright‐blood iT2Prep‐BOOST resulted in significantly improved image quality (mean ± standard deviation 3.8 ± 0.5 vs. 3.3 ± 0.8) and CR (2.9 ± 0.8 vs. 1.8 ± 0.5) compared with T2Prep‐bSSFP, with a shorter scan time (7.8 ± 1.7 minutes vs. 12.9 ± 3.4 minutes) while providing a complementary 3D black‐blood image. Aortic lumen diameter and vessel wall thickness measurements in bright‐blood and black‐blood images were in good agreement with T2Prep‐bSSFP and HASTE images (<0.02 cm and <0.005 cm bias, respectively) and good intrareader (ICC > 0.96) and interreader (ICC > 0.94) agreement was observed for all measurements.

**Data Conclusion:**

iT2Prep‐BOOST might enable time‐efficient simultaneous bright‐ and black‐blood aortic imaging, with improved image quality compared to T2Prep‐bSSFP and HASTE imaging, and comparable measurements for aortic wall and lumen dimensions.

**Evidence Level:**

2.

**Technical Efficacy:**

Stage 2.

Vascular MRI has emerged as an important imaging tool for the assessment of aortic disease over the last decade, related to the absence of ionizing radiation exposure and high sensitivity and specificity, and superior soft tissue contrast compared to alternative imaging modalities such as computed tomography (CT) and transthoracic echocardiography.^1^ Recently, published clinical guidelines proposed the acquisition of both bright‐blood and black‐blood MRI for the comprehensive assessment of aortic disease.^2^ Accurate aortic diameter measurements and rigorous, systematic documentation of luminal diameter changes over time are relevant for all patients with aortic pathology.^3^, ^4^ Additionally, T1‐weighted black‐blood imaging is required to enable high‐quality imaging of the aortic wall, and the measurement of arterial wall thickness in different vascular territories.^5^, ^6^, ^7^, ^8^, ^9^, ^10^ This has been shown to be clinically useful in the depiction of aortic dissection^5^ and in various research applications, including the prediction of adverse events in the presence of atherosclerosis,^6^, ^7^ anatomic description and biomechanical modeling in aneurysmal aortic disease,^8^, ^9^ and for prognostication after repair of coarctation of the aorta.^10^ Acquisition efficiency is also a requisite, as patients with aortic disease may undergo frequent and life‐long scanning. A novel sequence combining bright‐blood and black‐blood imaging and operating in short scan time is therefore clinically desirable for the comprehensive diagnosis and surveillance of complex aortic pathologies.

Conventionally, these two contrasts are acquired in separate scans. Bright‐blood images are usually acquired under free‐breathing using three‐dimensional (3D) contrast‐enhanced (CE) or non‐CE magnetization‐prepared MR angiography sequences that rely on diaphragmatic navigators to minimize the effect of respiratory motion and enable multiplanar evaluation of the luminal thoracic aorta along with the underlying vascular anatomy. However, the diaphragmatic navigator gating technique may result in long and unpredictable scan times or even failed scans in subjects with irregular breathing patterns. Several advanced respiratory motion compensation methods have been introduced in the literature to provide 100% respiratory scan efficiency for non‐CE aortic MR angiography, reducing scan time to 3–8 minutes, depending on the image resolution acquired. For instance, a self‐navigated sequence was used to acquire images of the aorta with a 1.1 mm isotropic resolution in 6.5 minutes.^11^, ^12^, ^13^ However, a reduced field of view was used to decrease total acquisition time, and therefore an additional scan was required when assessing the thoracic and abdominal aorta.^11^ To further remove respiratory‐induced blurring, a self‐navigated sequence was combined with a respiratory‐resolved reconstruction (XD‐GRASP), resulting in improved image quality compared to self‐navigation alone, enabling whole thoracic aortic imaging with a spatial resolution of 1.7 mm isotropic from a scan with 6 minutes.^14^, ^15^ More recently, a highly efficient approach for aortic MR angiography in 3 minutes has been introduced, with a 1.6 mm isotropic resolution.^16^ Using a Cartesian trajectory with spiral profile order with 3‐fold acceleration factor and combined with an image‐navigator approach to enable nonrigid respiratory motion correction, images of comparable quality to conventional diaphragmatic navigator gating were obtained, but in a fraction of the scan time.^16^

On the other hand, black‐blood imaging is generally acquired under breath‐hold utilizing two‐dimensional (2D) spin‐echo sequences in different orientations. This approach is utilized for evaluation of the aortic wall for hematoma or other causes of thickening, along with the quantification of plaque burden and detection of thrombus.^1^, ^2^ Conventional 2D black‐blood techniques rely on blood flow to null the blood signal, limiting their application for imaging the thoracic and abdominal aorta, and making planning challenging and time consuming. More recently, 3D imaging techniques have been proposed in the literature to enable black‐blood imaging with higher spatial resolution and improved spatial coverage.^17^, ^18^, ^19^, ^20^, ^21^, ^22^ These approaches use preparation pulses, including motion‐sensitized driven equilibrium (MDSE)^17^ and delay alternating with nutation for tailored excitation (DANTE)^18^; variable flip angle acquisitions,^19^, ^20^ or a combination of the last two^21^, ^22^ to produce black‐blood images of the thoracic and/or abdominal aorta. These approaches require between approximately 4 and 11 minutes to produce images with appropriate spatial resolution to visualize the aortic wall (1.2–1.3 mm isotropic), but to minimize respiratory‐induced artifacts they either rely on signal averaging, resulting in blurred images,^17^, ^20^, ^21^, ^22^ or use diaphragmatic navigator gating which leads to longer scan times.^19^ Furthermore, the separate scans for each contrast can result in potential misregistration between bright‐ and black‐blood images due to both patient motion and the different geometries and spatial resolution used for each scan.

In this study, we aimed to investigate an alternative technique for non‐CE simultaneous lumen and vessel wall imaging of the thoracic aorta,^23^ to achieve bright‐ and black‐blood contrast in shorter acquisition time than the current clinical standard.

## Materials and Methods

This study was performed in accordance with the Declaration of Helsinki and approved by the National Research Ethics Service (REC 15/NS/0030). Written informed consent was obtained from each participant according to institutional guidelines.

### Study Design and Population

This was a prospective monocentric study. Thirty consecutive patients (9 females, mean age ± standard deviation: 32 ± 12 years, min 19, max 61 years) with thoracic aortopathy referred for a clinical cardiac MRI examination were recruited for this study between August 2021 and February 2022. The diagnosis of aortopathy was described in the clinical referral form and 3D aortic imaging was part of the clinical request. Patients were eligible to participate if they were >18 years of age and consented to the study. Specific exclusion criteria were contraindications for cardiac MRI (eg pacemaker, cochlear implants, cerebral aneurysm clip, implanted electronic device, or claustrophobia) and inability to lie flat. Details about the patient cohort and corresponding diagnoses are presented in Supplementary Table S1. The investigated pathologies included inherited aortopathies (Marfan's syndrome), aortopathies due to complex congenital heart disease and congenital anomalies of the aortic valve and the thoracic aorta.

### MRI Framework

**FIGURE 1:**
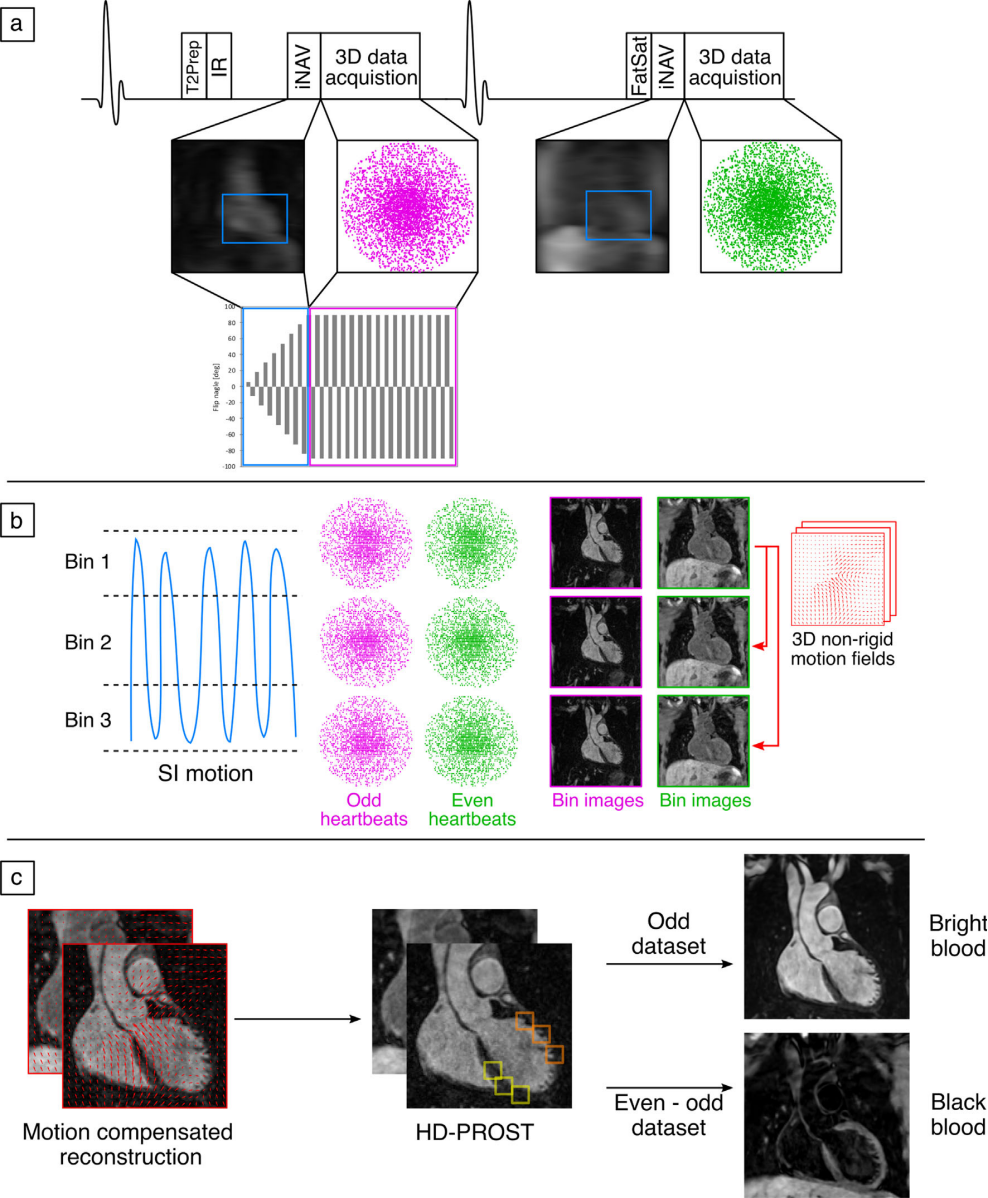
Proposed iT2Prep‐BOOST acquisition and reconstruction scheme. (a) 3D data acquisition is performed in an interleaved fashion, with a combined T2‐preparation and inversion recovery (T2Prep‐IR) preparation pulse used in odd heartbeats, and only fat saturation used in even heartbeats. The sequence integrates image navigators (iNAVs) for motion compensation and 100% respiratory scan efficiency. (b) Superior inferior (SI) motion estimated from the iNAVs is used to bin the data and produce respiratory‐resolved 3D bin images, which are then used to estimate 3D nonrigid motion. (c) Co‐registered motion‐corrected 3D images are obtained by incorporating nonrigid motion into the reconstruction and low‐rank patch‐based denoising, which are finally used to obtain the bright‐blood data for lumen visualization (odd heartbeats) and black‐blood data for vessel wall visualization (by direct subtraction of even and odd heartbeats).

Data acquisition with the proposed sequence (iT2Prep‐BOOST) consisted of an electrocardiogram (ECG)‐triggered interleaved 3D balanced steady‐state free precession (bSSFP) sequence (Figure 1a), where a combined T2 preparation and inversion recovery (T2Prep‐IR) preparation module was applied before data acquisition in odd heartbeats and fat saturation was applied in even heartbeats, resulting in two bright‐blood datasets. The 2D image navigators (iNAVs)^24^ were acquired at each heartbeat by spatially encoding the start‐up echoes of the bSSFP acquisition to enable 100% respiratory efficiency (without data rejection) and predictable scan time. To further reduce scan time, 3D data were acquired with an undersampled variable‐density golden‐step Cartesian trajectory with spiral profile order sampling.^25^, ^26^

The iNAVs were used to estimate the respiratory signal, so that by tracking a rectangular template located around the aortic arch, translational superior–inferior (SI) and right–left (RL) respiratory motion can be obtained.^24^ The SI and RL respiratory motions were used to produce nonrigid motion‐compensated bright‐blood volumes as previously described.^23^, ^27^ Briefly, the SI motion was used to group the acquired data into equally populated respiratory bins, and binned 3D data were corrected for rigid SI and RL respiratory motion to the center of the corresponding bin. Respiratory‐resolved bin images were then reconstructed with iterative sensitivity encoding (SENSE) at half of the full imaging resolution, histogram‐equalized, and subsequently used to estimate the non‐rigid bin‐to‐bin respiratory motion fields, using the end expiratory bin as the reference position (Figure 1b).

The nonrigid deformation fields were then interpolated back to full image resolution and were incorporated into a generalized matrix formulation for motion‐compensated MR reconstruction^28^ This was done to obtain two co‐registered motion‐compensated bright‐blood datasets. To minimize blurring and artifacts arising from the undersampled acquisition, a low‐rank patch‐based denoising (High‐Dimensional Patch‐based RecOnStrucTion, HD‐PROST) was implemented.^29^ Finally, the first bright‐blood dataset can be applied for lumen visualization, while digital subtraction of the two bright‐blood datasets was used to create the primarily T1‐weighted black‐blood dataset for vessel wall visualization (Fig. 1c). The described motion‐compensated reconstruction including respiratory binning and nonrigid motion correction was performed in‐line on the scanner without any regularization, while low‐rank patch‐based denoising was performed offline in MATLAB (The MathWorks, Natick, MA).

### Experiments

The proposed framework was implemented as a prototype on a 1.5‐T MRI system (MAGNETOM Aera, Siemens Healthcare, Erlangen, Germany). All acquisitions were performed on this system using an 18‐channel chest‐coil and a 32‐channel spine coil.

The clinical protocol included a standard non‐CE bright‐blood 3D MR angiography, acquired with the following sequence parameters: T2‐prepared bSSFP readout, sagittal orientation, field of view (FOV) = 400 × 212–300 × 134–202 mm^3^, spatial resolution = 1.4 mm^3^ isotropic, T2Prep duration = 40 ms, GeneRalized Autocalibrating Partial Parallel Acquisition (GRAPPA) parallel imaging 2× undersampled, flip angle = 90°, repetition time (TR)/echo time (TE) = 3.5/1.7 msec, bandwidth = 930 Hz/px. These acquisitions were performed under free breathing, with diaphragmatic navigator gating for respiratory motion compensation, with a gating window of ±3.5 mm in end‐expiration and a slice‐tracking factor of 0.6.

In order to visualize the aortic wall, patients also underwent 2D breath‐hold black‐blood half acquisition single‐shot turbo spin echo (HASTE) imaging with the following parameters: axial orientation, in‐plane resolution = 1.56 × 1.56 mm^2^, slice thickness = 8 mm, TR/TE = 3/40 msec, flip angle = 160°, partial Fourier = 5/8, bandwidth = 780 Hz/px, turbo factor = 104, 23–36 slices acquired in 3–5 breath‐holds, with additional orientations acquired depending on each patients' diagnosis.

After the clinical scan, data were acquired with the proposed 3D iT2Prep‐BOOST method, with the following parameters: bSSFP readout, coronal orientation, FOV = 400 × 300 × 104–208 mm^3^, spatial resolution = 1.3 mm^3^ isotropic, 4 × undersampling, flip angle = 90°, TE/TR = 1.41/3.24 msec, bandwidth = 965 Hz/px. In odd heartbeats, a T2Prep‐IR preparation module was used, with a T2Prep duration of 40 msec, and inversion time (TI) = 110 msec, while in even heartbeats only fat saturation was used. For both the clinical 3D T2Prep and the proposed 3D iT2Prep‐BOOST acquisitions, a subject‐specific trigger delay and acquisition window (120–150 msec) were set to coincide with ventricular mid‐diastole to minimize the effect of cardiac motion. These acquisition parameters were estimated by visually inspecting a free‐breathing 4‐chamber cine image. Scan time was recorded for all sequences. In‐line reconstruction time and off‐line denoising for the iT2prep‐BOOST was also recorded.

### Data Analysis

#### VISUAL ANALYSIS

Image quality of the proposed iT2Prep‐BOOST bright‐blood images and conventional T2Prep‐bSSFP bright‐blood images was assessed by three cardiologists (R.H., A.F., and A.H.) with 4, 3, and 3 years of experience in cardiovascular MRI, respectively (R.H. and A.F: level III accreditation in cardiac MRI). In total, 60 sets were randomized and de‐identified for display on a 3D workstation (Osirix, version 9.0; OsiriX Foundation, Geneva, Switzerland).

Before visual evaluation, the readers were given training datasets with poor to excellent image quality to calibrate their scores together. Training datasets for the corresponding image quality scores have been demonstrated in a previous study.^16^ Following this training session, each reader was blinded to the image acquisition type, reconstruction method, the other readers' evaluations, and clinical history for independent evaluation. Image quality assessment was based on a 4‐point scoring system and included sharpness of the vessel (1 = nondiagnostic, 2 = poor, 3 = adequate to good, and 4 = excellent) and presence of image artifacts (1 = severe artifact, 2 = moderate artifact, 3 = mild artifact, and 4 = minimal artifact) at four predefined anatomical locations: aortic root (ARoot), mid ascending aorta (mAA), mid aortic arch (mAAr), and mid descending aorta (mDA).

The readers also assessed overall diagnostic confidence of the iT2Prep‐BOOST and the T2Prep‐bSSFP images, considering 1) exclusion of diseases, 2) diagnosis of suspected abnormalities, and 3) diagnosis of unsuspected abnormalities relevant to the patients' assessment regarding aortopathy. A 4‐point score system was used to assess the level of confidence, with 1 = poor image quality, poorly defined anatomic details, poor diagnostic confidence; 2 = reduced image quality, limitations in anatomic detail, impairment of diagnostic confidence; 3 = good image quality, clear anatomic details, no impairment of diagnostic confidence; and 4 = excellent image quality, distinct anatomic details, full diagnostic confidence.

#### QUANTITATIVE IMAGE QUALITY ANALYSIS

Contrast between aortic lumen and myocardium was computed in the conventional T2Prep‐bSSFP and proposed iT2prep‐BOOST bright‐blood images to quantify image quality. For this, spherical regions of interest (ROIs) were manually placed by reader 2 (A.F.) in the left ventricular myocardium at mid‐ventricular level (sphere of 5 mm diameter) and the aortic blood pool (sphere of 15 mm diameter) at the same anatomical locations (ARoot, mAA, mAAr, mDA) using Osirix (version 9.0; OsiriX Foundation). Blood‐to‐myocardium contrast ratio (CR) was computed at each location as

$$\frac{\left(\mu_{\mathrm{ROI},\text{lumen}}-\mu_{\mathrm{ROI},\text{myocardium}}\right)}{\mu_{\mathrm{ROI},\text{myocardium}}}$$

where $\mu_{\mathrm{ROI}}$ corresponded to the mean value in each ROI.

For the black‐blood images, contrast ratio between vessel wall and blood pool was also calculated. For this, reader 2 manually placed an ROI in the vessel wall and plod pool at the level of the mDA using Osirix (version 9.0; OsiriX Foundation). The same ROIs were used for both HASTE and iT2Prep‐BOOST black blood images, and the vessel wall‐to‐blood pool was computed as

$$\frac{\left(\mu_{\mathrm{ROI},\text{vessel wall}}-\mu_{\mathrm{ROI},\text{blood pool}}\right)}{\mu_{\mathrm{ROI},\text{blood pool}}}$$

#### AORTIC DIAMETER MEASUREMENTS

Additionally, aortic diameter was measured in the bright‐blood images at three landmarks (ARoot, mAA, mDA). Anatomic landmarks were used to reformat the bright‐blood volumes and find appropriate views. Measurements were performed perpendicular to the longitudinal axis of the aorta to correct for the variable geometry of the aorta. Blinded co‐axial measurements (maximum diameter) were performed using high‐resolution multiplanar reformats (Osirix, version 9.0; OsiriX Foundation) by readers 2 and 3 (A.H.) for interobserver reliability assessment. Reader 2 repeated the measurements in the iT2Prep‐BOOST dataset twice to assess intrareader reliability.

#### AORTIC WALL THICKNESS MEASUREMENTS

Vessel wall thickness measurements were performed in the black‐blood iT2Prep‐BOOST and HASTE images at the level of the mAA following a method proposed previously.^30^, ^31^ In short, ROIs were manually delineated by reader 2 to include the internal and external wall of the aorta, so that a luminal aortic area and total aortic area were obtained. These areas were approximated as circles; the radius (r) of each area (A) was calculated according to the following formula: r = √(A/π). Finally, mean aortic wall thickness (MAWT) was calculated as the difference of the radii of the external and internal area, separately for the ascending and the descending aorta.

#### CHRONIC AORTIC DISSECTION iT2Prep ‐BOOST vs. CT

One distinct case with chronic aortic dissection that was followed‐up with 6‐monthly clinically indicated CT scans underwent a focused pilot MRI scan with the proposed sequence. The individual iT2Prep‐BOOST aortic scan was visually compared with the corresponding clinically indicated CT scan.

### Statistical Analysis

Statistical analysis was performed using SPSS (version 26.0.0.1, Statistical Package for the Social Sciences; IBM Inc., Armonk, NY, USA) and Prism (version 9.1.0; GraphPad Software, San Diego, CA, USA). Categorical variables are presented as frequencies and corresponding percentages. Results are expressed as mean ± standard deviation (SD, if normally distributed) or as median with interquartile range (IQR, if not normally distributed).

Total scan time for the clinical approach (T2prep‐bSSFP and HASTE) and the proposed iT2Prep‐BOOST method, blood‐to‐myocardium CR in the bright‐blood images and vessel wall‐to‐blood pool CR in the black‐blood images were compared with a paired two‐tailed Student's *t*‐test to assess statistically significant differences. Image quality scores of the iT2Prep‐BOOST and conventional T2Prep‐bSSFP imaging techniques (including vessel sharpness, presence of artifacts, and overall diagnostic confidence) were compared for each reader using Wilcoxon signed‐rank tests.

Bland–Altman plots were used to assess agreement of measurements of aortic diameter in bright‐blood images using both techniques and intrareader agreement for the proposed iT2Prep‐BOOST. Intraclass correlation coefficients (ICCs) were calculated to determine the interreader and intrareader concordance. Specifically, ICC estimates and their 95% confidence intervals (CIs) were calculated based on two‐way mixed effects models to define the interreader and intrareader reliability. For assessment of measurements of vessel wall thickness comparing the black‐blood iT2Prep‐BOOST and HASTE images, Bland–Altman analysis and linear correlations were used. For all statistical tests, two‐tailed *P*‐values <0.05 were considered statistically significant.

## Results

Imaging was successfully completed in all 30 patients. The scan time for the proposed iT2Prep‐BOOST was significantly shorter than that for the clinical sequences, including T2Prep‐bSSFP and HASTE (7.8 ± 1.7 minutes vs. 12.9 ± 3.4 minutes). In‐line reconstruction time for the iT2prep‐BOOST was 3 ± 0.3 minutes and off‐line denoising was 4 ± 0.5 minutes.

### Visual Analysis

**FIGURE 2:**
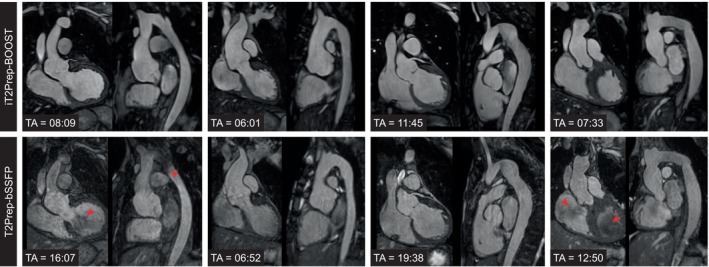
Visual comparison between bright‐blood images produced by the proposed iT2Prep‐BOOST approach compared to the conventional T2prep‐bSSFP for four representative patients (columns) showing a coronal view and a reformatted long axis view of the thoracic aorta. Acquisition time (TA) is expressed in minutes:seconds. (a) A 35‐year‐old patient with bicuspid aortic valve and coarctation of the aorta. Flow artefacts at the level of the arch, the isthmus, and the left ventricular outflow tract (red arrows) are reduced; (b) 19‐year‐old patient with aortic root aneurysm. Respiratory artefacts in the aortic root are reduced in the proposed approach; (c) 20‐year‐old patient with aortic aneurysm in the descending aorta, post coarctation repair with end‐to‐end anastomosis; (d) 43‐year‐old patient with tortuous thoracic aorta post coarctation repair. Blood pool inhomogeneities (red arrows) are attenuated.

Figure 2 shows a visual comparison between bright‐blood images produced by iT2Prep‐BOOST and the conventional T2Prep‐bSSFP sequence for four representative participants, including a coronal view and a reformatted long axis along the thoracic aorta. A reduction in respiratory‐induced and flow‐induced artifacts can be observed with the proposed method. There is increased visual contrast between blood pool and myocardium and improved blood signal homogeneity due to the T2Prep‐IR preparation pulse.

**FIGURE 3:**
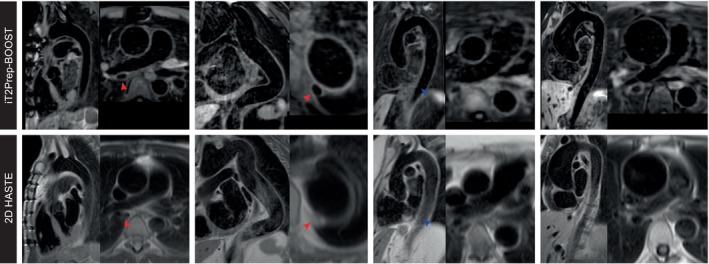
Visual comparison between black‐blood images produced by the proposed iT2Prep‐BOOST approach compared to the conventional 2D HASTE for four representative patients (columns) showing a long axis view and a transverse view of the thoracic aorta. (a) A 31‐year‐old patient with aortic homograft in situ in view of bicuspid aortic valve disease. The aortic wall is demonstrated with improved signal in the proposed iT2prep‐BOOST. (b) Tortuous thoracic aorta in a 42‐year‐old patient with Marfan's syndrome post mechanical aortic valve replacement. (c) Thoracic aortic in a 62‐year‐old patient with Marfan's syndrome. (d) Aortic aneurysm in a 37‐year‐old patient with bicuspid aortic valve.

Black‐blood images obtained with iT2Prep‐BOOST compared to the conventional 2D HASTE images are shown in Fig. 3 for additional four participants, including a reformatted long‐axis view and a transverse view of the thoracic aorta. While each 2D HASTE image is acquired in a separate breath‐held scan, with limited coverage and nonisotropic resolution, iT2Prep‐BOOST black‐blood images cover the whole thoracic aorta and can be reformatted into any plane of interest. Image quality can be degraded by insufficient blood suppression in the sagittal oblique views of the 2D HASTE, due to the complex flow patterns in the tortuous descending aorta. Images acquired with iT2prep‐BOOST demonstrated consistent blood suppression that was independent of the direction of blood flow. Furthermore, the increased spatial resolution of the proposed 3D approach improved the depiction of the vessel wall.

For all three readers, overall diagnostic confidence based on the bright‐blood images was higher for the proposed iT2Prep‐BOOST sequence than for the conventional T2Prep‐bSSFP sequence, reaching statistical significance for two of the readers (R1: 4 (4) vs. 3 (3); R2: 4 (4) vs. 4 (3, 4), *P* = 0.0625; R3: 4 (4) vs. 4 (3, 4)). For the iT2Prep‐BOOST method, one dataset was deemed as nondiagnostic for two of the readers, in view of significant artifacts from an adjacent left pulmonary artery stent, while for the T2Prep‐bSSFP, three, one, and two datasets were deemed nondiagnostic for readers 1, 2, and 3, respectively, which was due to respiratory artifacts, flow‐related artifacts, and artifacts from a stent for reader 1, left pulmonary artery stent artifacts for reader 2, and artifacts from a left pulmonary artery stent and flow‐related artifacts for reader 3.

**FIGURE 4:**
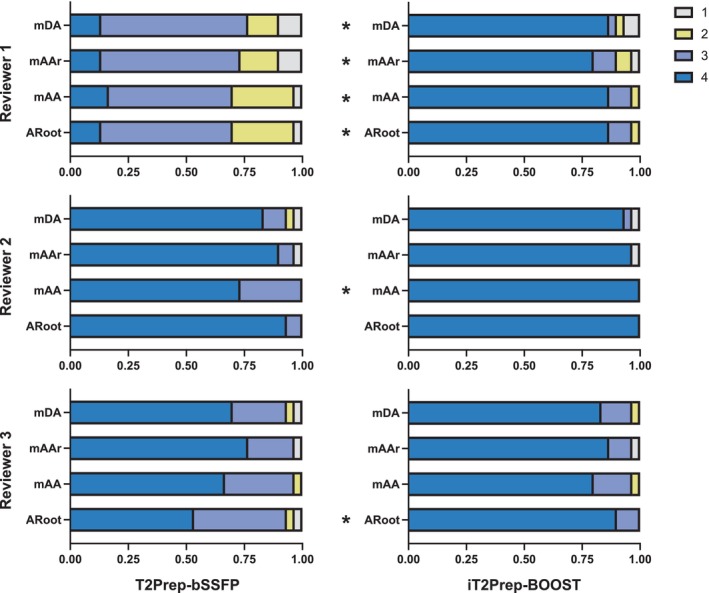
Image quality scores analysis with respect to sharpness of the vessel (1 = nondiagnostic, 4 = excellent) for the proposed iT2Prep‐BOOST (right) in comparison to the clinical T2Prep‐bSSFP (left) bright‐blood images. Scores for the three reviewers are shown, at the mid descending aorta (mDA), mid aortic arch (mAAr), mid ascending aorta (mAA) and aortic root (ARoot) levels. *indicates that iT2Prep‐BOOST quality scores are significantly higher than T2Prep‐bSSFP scores, in the other cases the difference was not statistically significant.

**FIGURE 5:**
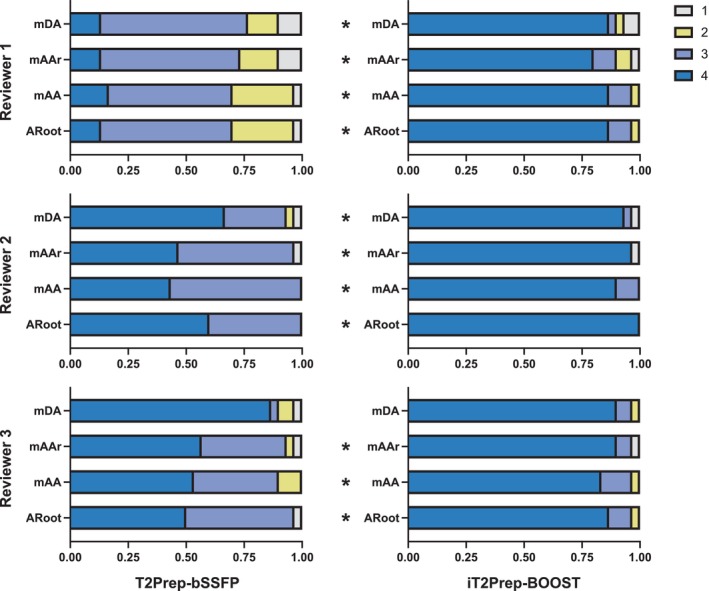
Image quality scores analysis with respect to presence of artifacts in the images (1 = severe artifact, 4 = minimal artifact) for the proposed iT2Prep‐BOOST (right) in comparison to the clinical T2Prep‐bSSFP (left) bright‐blood images. Scores for the three reviewers are shown, at the mid descending aorta (mDA), mid aortic arch (mAAr), mid ascending aorta (mAA) and aortic root (ARoot) levels. *indicates that iT2Prep‐BOOST quality scores are significantly higher than T2Prep‐bSSFP scores, in the other cases the difference is not statistically significant.

Summary results of the image quality assessment considering sharpness of the vessel and presence of artifacts at the ARoot, mAA, mAAr, and mDA are shown in Figs. 4 and 5, respectively. Specifically, iT2Prep‐BOOST resulted in improved image quality compared to T2Prep‐bSSFP bright‐blood images when comparing visual vessel sharpness, reaching statistical significance for two Readers at the ARoot (readers 1 and 3) and the mAA (readers 1 and 2), and for one reader at the mAAr and mDA (reader 1). On the other hand, statistically significant improvements regarding presence of artifacts were observed for all three readers at the ARoot, mAA and mAAr, and for two readers at the mDA (readers 1 and 2). Detailed values can be found in Supplementary Tables S2 and S3.

### Quantitative Image Quality Analysis

**FIGURE 6:**
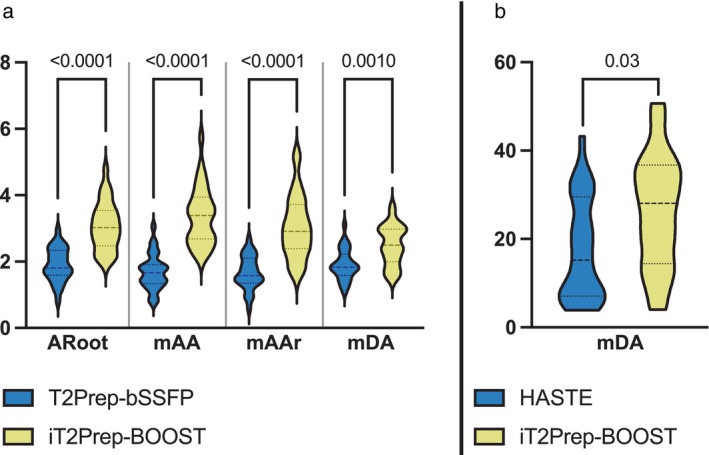
(a) Contrast ratio (CR) between blood‐pool at the mid descending aorta (mDA), mid aortic arch (mAAr), mid ascending aorta (mAA) and aortic root (Aroot) levels and myocardium for the proposed iT2Prep‐BOOST (in yellow) in comparison to the clinical T2Prep‐bSSFP (in blue) bright‐blood images. (b) CR between vessel wall and blood pool at the mDA level for the proposed iT2Prep‐BOOST (in yellow) in comparison to clinical HASTE (in blue) black blood images. The proposed iT2Prep‐BOOST method results in significantly higher CR compared to the conventional approaches for both bright and black blood images, with *P* values as indicated in the figure.

Lumen‐to‐myocardium CR was significantly higher for the iT2Prep‐BOOST approach compared to the conventional T2Prep‐bSSFP at all aortic levels (Fig. 6a). The average CR at the ARoot was 3.0 ± 0.7 vs. 1.9 ± 0.5, at the mAA was 3.3 ± 0.8 vs. 1.6 ± 0.5, at the mAAr was 3.0 ± 0.9 vs. 1.6 ± 0.5, and at the mDA was 2.5 ± 0.6 vs. 1.9 ± 0.4.

Similarly, in the black‐blood images, vessel wall‐to‐blood pool CR was significantly higher for the iT2Prep‐BOOST approach compared to HASTE images 25.9 ± 12.9 vs. 17.7 ± 11.6 (Fig. 6b).

### Aortic Diameter Measurements

**FIGURE 7:**
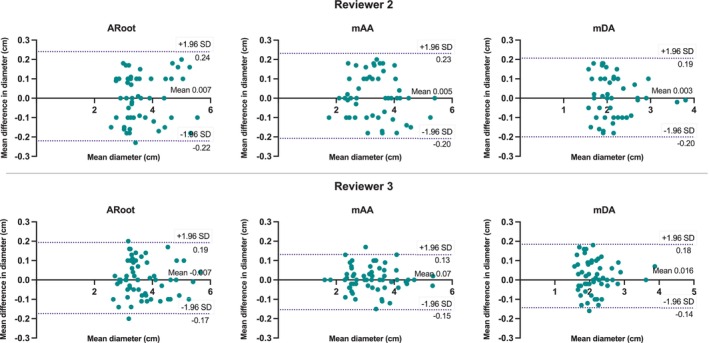
Bland–Altman analysis for co‐axial diameter measurements at the aortic root (ARoot), mid ascending aorta (mAA), and mid descending aorta (mDA), comparing the iT2Prep‐BOOST and T2prep‐bSSFP bright‐blood images for reviewer 2 (top row) and reviewer 3 (bottom row). In each plot, the solid black line indicates the mean bias of the diameter measurements, whereas the dotted lines represent the 95% confidence interval. Values are given in centimeter.

Bland–Altman analysis for the results on aortic diameter measurements from bright‐blood images is shown in Fig. 7 and Supplementary Table S4, demonstrating good agreement between measurements. Limits of agreement were between (−2.2 mm and +2.4 mm) for both readers.

**FIGURE 8:**
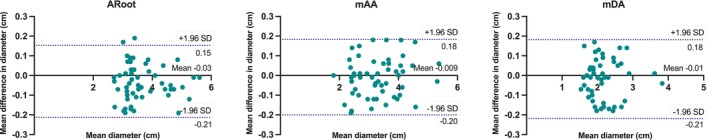
Bland–Altman analysis for intrarater co‐axial aortic diameter measurements at the aortic root (ARoot), mid ascending aorta (mAA) and mid descending aorta (mDA), for the iT2Prep‐BOOST images. Measurements were performed twice by reviewer 2. In each plot, the solid black line indicates the mean bias of the diameter measurements whereas the dotted lines represent the 95% confidence interval. Values are given in centimeter.

Good intrareader agreement was observed for the bright‐blood iT2Prep‐BOOST measurements, with limits of agreement between −2.1 mm and +1.8 mm (Fig. 8). Detailed limits of agreement at different anatomical positions are shown in Supplementary Table S5. Furthermore, excellent intrareader (ICC > 0.96) and interreader (ICC > 0.94) agreement was observed for all measurements (Supplementary Table S6) for images acquired with the proposed iT2Prep‐BOOST method.

### Aortic Wall Thickness Measurements

**FIGURE 9:**
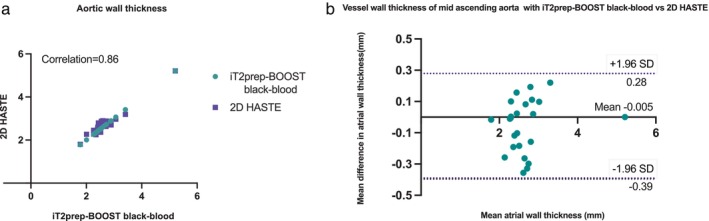
(a) Correlation of aortic wall thickness with black‐blood iT2prep‐BOOST vs. 2D HASTE. (b) Bland–Altman analysis for aortic wall thickness measurements at the level of the mid ascending aorta with iT2Prep‐BOOST vs. 2D HASTE. The solid black line indicates the mean bias of the vessel wall measurements, whereas the dotted lines represent the 95% confidence interval. Values are given in millimetre. (c) Intrarater reproducibility of aortic wall thickness with black‐blood iT2prep‐BOOST. (d) Bland–Altman analysis for aortic wall thickness intrarater reproducibility measurements at the level of the mid ascending aorta with iT2Prep‐BOOST. The solid black line indicates the mean bias of the vessel wall measurements, whereas the dotted lines represent the 95% confidence interval. Values are given in millimetre.

Good agreement between measurements of wall thickness from black‐blood images can be observed in the Bland–Altman analysis (Fig. 9), with limits of agreement of −0.4 and +0.3 mm, and a correlation with *R* = 0.86.

### Chronic Aortic Dissection iT2prep‐BOOST vs. CT

**FIGURE 10:**
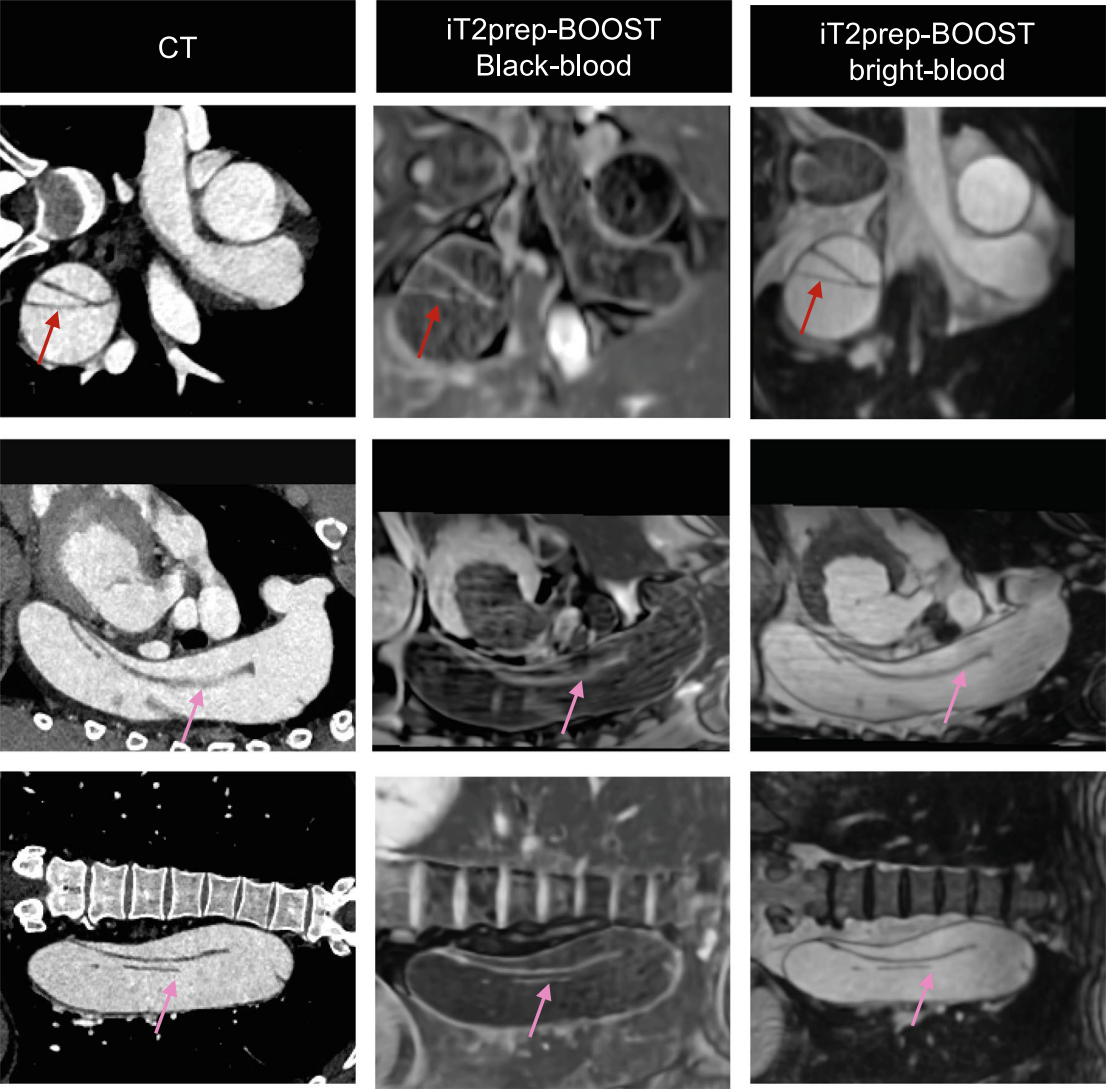
Comparison between conventional CT and proposed iT2Prep‐BOOST MR imaging in 54‐year‐old patient with chronic aortic dissection. The dissection flap is complex and extends from the aneurysmal thoracic descending aorta to the left common iliac artery (pink arrow). It is demonstrated both in the proposed iT2prep‐BOOST and the CT images (red arrow). The false lumen is dilated and aneurysmal.

Figure 10 shows images from the additional patient with chronic aortic dissection type B. The sagittal reformat shows the extension of the intimal flap into the descending aorta with comparable image quality between the iT2prep‐BOOST and the clinical CT scan. Aortic cobwebs in the false lumen are shown in both the CT and the iT2prep‐BOOST images.

## Discussion

This study demonstrated the feasibility of a motion‐corrected 3D interleaved T2Prep‐IR sequence (iT2Prep‐BOOST) for simultaneous bright‐ and black‐blood imaging of the thoracic aorta in patients with aortopathy. To assess the performance of iT2Prep‐BOOST, bright‐blood images were compared to conventional T2prep‐bSSFP images for the visualization of cardiac and vascular anatomy, while black‐blood images were compared to 2D HASTE imaging for visualization of the aortic vessel wall. Results showed very good agreement between the proposed and routinely used T2Prep‐bSSFP and HASTE techniques in terms of aortic vessel diameter and wall thickness, respectively; however, the proposed bright‐blood iT2Prep‐BOOST achieved images with higher image quality scores and overall diagnostic confidence and improved lumen‐to‐myocardium CR. Furthermore, the iT2Prep‐BOOST provided free‐breathing black‐blood images with higher isotropic spatial resolution and volumetric coverage, and with reduced and more predictable scan times. This could be relevant for patient comfort, cooperation, and therefore future compliance.

The reduction in scan time might arise both from the 4‐fold undersampled acquisition (compared with the 2‐fold accelerated GRAPPA used for the conventional 3D T2prep‐bSSFP) and the use of image‐based navigation and a nonrigid motion‐corrected image reconstruction approach that enables 100% respiratory scan efficiency. Conventional 3D cardiac imaging relies on the use of diaphragmatic navigator gating to minimize the effect of respiratory motion. While this approach produces good‐quality images in subjects with regular breathing patterns, it may result in long‐scan times and poor‐quality images in subjects with irregular breathing patterns. Indeed, previous studies have reported between 8% and 14% failed/nondiagnostic scans due to irregular breathing.^32^, ^33^ The proposed iT2Prep‐BOOST approach on the other hand corrects for the nonrigid respiratory motion of the aorta during the scan, resulting in more predictable acquisition times and fewer artifacts.

A significantly higher contrast between aortic lumen and myocardium was observed for the bright‐blood iT2Prep‐BOOST images compared to conventional T2prep‐bSSFP, which was potentially due to the use of the combined IR and T2 preparation pulses as previously shown in the literature.^34^, ^35^ While these methods enable the assessment of the lumen of the vessel, they do not provide means for assessing the vessel wall. More recently, multicontrast approaches that enable simultaneous bright‐ and black‐blood imaging of the aorta have been introduced in the literature.^36^, ^37^ Hu et al introduced MT‐MACS, a multitasking‐based approach for multidimensional assessment of the cardiovascular system, which enables bright‐, black‐, and gray‐blood imaging with 1.38 mm^3^ isotropic resolution from a single scan with 6 minutes, albeit requiring a long reconstruction time of 110 minutes.^36^ Tachikawa et al proposed bright and dark blood images with multishot gradient‐echo EPI (BRIDGE), achieving good quality black‐ and bright‐blood images with a nonisotropic resolution of 1.6 × 1.6 × 3 mm from a scan with ~3.5 minutes.^37^ While promising, these approaches have mainly been demonstrated in healthy subjects or small cohorts of patients (≤10).^36^, ^37^ On the other hand, in this study, iT2Prep‐BOOST has been applied to a cohort of 30 patients with aortopathy, obtaining high‐resolution isotropic images (1.3 mm^3^) from a scan with ~8 minutes that requires ~3 minutes in‐line image reconstruction time and 3‐5 minutes offline denoising, thus potentially encouraging a more straightforward adoption in the clinical routine.

The iT2Prep‐BOOST sequence presented in this article uses a bSSFP readout, which might be susceptible to artifacts induced by field inhomogeneities at higher magnetic fields. In order to be used at 3 T, a modified version of this approach with a Dixon gradient echo readout could provide improved robustness against field inhomogeneities and reliable fat suppression.

The proposed method may accurately delineate lumen along with the endocardial border of the heart and the lumen‐wall interface of vessels. The primarily T1‐weighted black‐blood images could thus be promising for the depiction of atheroma, intramural hematomas, thrombi, and therefore further validation in patients with atherosclerosis, chronic aortic dissection with concurrent hemorrhage, and/or thrombosis is warranted. Further applications of this technique could include diseases where high definition of the cardiac wall and myocardium is required, such as noncompaction cardiomyopathy and atrial wall imaging in electrophysiology. Further, 3D virtual auto‐focus navigation could be a promising alternative to alleviate residual respiratory motion‐induced artifacts and further enhance image quality.^38^ Deep learning‐based reconstruction methods could further accelerate the acquisition time and potentially the clinical workflow, helping to introduce this modality in acute aortic dissection imaging as well.

### Limitations

The current study included a limited number of subjects and a larger study would allow for inclusion of a broader representation of patients with aortopathy. There was a slight discrepancy in the resolution between the clinical protocol (1.4 mm^3^ bright‐blood T2prep‐bSSFP and 1.56 mm^2^ in plane resolution for 2D HASTE) and the proposed approach. The clinical protocol was determined by the clinical team and could not be altered. The proposed sequence, aiming to offer a novel approach to thoracic aortic imaging, was implemented at a higher resolution (1.3 mm^3^), which was done to enable sharp delineation of both the lumen and the wall. The accelerated acquisition scheme made this possible in an overall shorter acquisition time, which is however still susceptible to heart rate variations.

While a comparison between conventional and proposed bright‐blood images in terms of image quality and diagnostic confidence was performed, we did not perform such a comparison for black‐blood images. iT2prep‐BOOST offers a 3D visualization of the aorta and can be retrospectively reformatted into any view of interest, which makes it inherently superior to the 2D HASTE which has a limited number of views. In future, comparison with a reference 3D black‐blood sequence could be performed to further characterize image quality and diagnostic confidence for the proposed method. Furthermore, the clinical 2D HASTE sequence had lower spatial resolution and larger slice thickness compared to the iT2prep‐BOOST black‐blood images, which may have biased measurements. It is also worth noting that currently there is no reference standard method for the measurement of mean aortic wall thickness. While previous studies demonstrated that the vessel‐area method might introduce the least amount of error in the determination of mean aortic wall thickness,^30^, ^31^ further validation against CT imaging or histology is required.

Residual respiratory artifacts in the anteroposterior direction were observed in some datasets. The underlying contrast between the blood pool and the vessel wall in the dark‐blood datasets renders the respiratory motion‐related ghosting artifacts more visible within the aortic lumen.^38^ The proposed black‐blood dataset is flow‐independent and does not null the signal from stents or devices, contrary to the black‐blood spin echo sequences. However, neither spin echo nor black‐blood iT2prep‐IR is the optimal means to quantify the degree of stent stenosis, if any.^39^ Finally, this is a proof‐of principle study and our cohort included relatively young participants who were investigated for aortic aneurysms, and none of them was diagnosed with underlying arrhythmia.

## Conclusion

This study suggests that an iT2prep‐BOOST sequence may provide high‐quality bright‐ and black‐blood imaging for comprehensive assessment of the thoracic aorta. The technical feasibility of this method has been shown in a cohort of patients with uncomplicated aortopathy and in one patient with chronic aortic dissection.

## Supporting information


**Appendix S1:** Supplementary Information
