# Supplementary material for: Simultaneous Highly Efficient Contrast‐Free Lumen and Vessel Wall MR Imaging for Anatomical Assessment of Aortic Disease
Source: J Magn Reson Imaging. 2023 Feb 9;58(4):1110–22. doi: 10.1002/jmri.28613 (PMC10946808; doi:10.1002/jmri.28613)
Supplement: Supplementary file 1 — Appendix S1: Supplementary Information [file JMRI-58-1110-s001.docx]

**Supplementary Tables**

Supplementary Table 1. Patient cohort and corresponding diagnosis and procedures.

|  | Gender | Age (y.o.) | Diagnosis & procedures | Height (cm) | Weight (kg) | HR (bpm) | Acquisition challenges |
| --- | --- | --- | --- | --- | --- | --- | --- |
| Patient 01 | M | 21 | Dilated aortic root.  Transposition of the great arteries  S/P: Arterial switch procedure with bicuspid pulmonary autograft. | 190 | 63 |  | Irregular breathing pattern |
| Patient 02 | M | 25 | Dilated aortic root. Tetralogy of Fallot  S/P: Tetralogy of Fallot repair | 185 | 87 |  |  |
| Patient 03 | F | 35 | Transposition of the great arteries.  Severely dilated aortic root.  Aorta compression due to left and right pulmonary artery stents.  S/P: Arterial switch procedure | 165 | 104 | 77 | High BMI. Pulmonary artery stents. |
| Patient 04 | M | 34 | Coarctation of the aorta. Bicuspid aortic valve.  S/P: Coarctation of the aorta repair with Gore-tex patch.  Ross procedure. | 180 | 72 | 66 | Irregular heart rate |
| Patient 05 | M | 34 | Coarctation of the aorta.  S/P: Subclavian flap repair.  Stenting of transverse arch and isthmus. | 176 | 74 | 53 | 2 aortic stents |
| Patient 06 | M | 29 | Bicuspid aortic valve.  Moderately dilated aortic root and ascending aorta. | 182 | 67 | 69 |  |
| Patient 07 | M | 30 | Bicuspid aortic valve.  Dilated ascending aorta.  S/P: Aortic valve replacement with aortic homograft. | 190 | 71 | 58 |  |
| Patient 08 | M | 20 | Coarctation of the aorta  S/P: Coarctation of the aorta repair with end-to-end anastomosis. | 180 | 77 | 89 | Tachycardic with irregular heart rate |
| Patient 09 | M | 20 | Bicuspid aortic valve.  Mild aortic root dilatation | 175 | 73 | 65 |  |
| Patient 10 | M | 39 | Coarctation of the aorta.  Mild aortic root dilatation.  Mild residual narrowing at the isthmus.  S/P: Repair with subclavian flap. | 180 | 74 | 82 |  |
| Patient 11 | M | 30 | Shone’s complex: coarctation of the aorta, abnormal mitral valve, bicuspid aortic valve, subaortic stenosis. Mildly dilated aortic root and ascending aorta.  S/P: Stent at the isthmus. | 152 | 66 | 62 | Stent |
| Patient 12 | F | 39 | Aortic stenosis  S/P: Ross procedure. | 1.60 | 85 | 63 |  |
| Patient 13 | F | 23 | Coarctation of the aorta. Mild residual narrowing at the isthmus. Bicuspid aortic valve.  Dilated aortic root, ascending aorta and mid descending aorta.  S/P: Coarctation of the aorta repair and reduction aortoplasty with personalized external root support procedure. | 1.83 | 70 | 74 |  |
| Patient 14 | M | 52 | Marfan syndrome.  Tortuous thoracic aorta with moderate dilatation.  S/P: Aortic valve replacement with mechanical valve. | 193 | 80 | 59 | Mechanical aortic valve |
| Patient 15 | M | 19 | Aortic stenosis. Aortic root aneurysm. Dilatation of the ascending aorta.  S/P: Ross procedure. | 175 | 67 | 73 |  |
| Patient 16 | M | 28 | Bicuspid aortic valve disease. | 189 | 105 | 59 | High BMI  Irregular breathing pattern |
| Patient 17 | M | 20 | Bicuspid aortic valve.  Aortic aneurysm.  Coarctation of the aorta repair with subclavian flap. | 193 | 89 | 60 |  |
| Patient 18 | F | 61 | Marfan’s syndrome. | 160 | 79 | 86 |  |
| Patient 19 | F | 19 | Hypoplastic aortic arch.  Subaortic stenosis. | 174 | 80 | 80 |  |
| Patient 20 | M | 19 | Marfan syndrome | 206 | 109 | 53 |  |
| Patient 21 | F | 37 | Bicuspid aortic valve with moderate regurgitation.  Aneurysm in the ascending aorta. | 178 | 87 | 65 |  |
| Patient 22 | M | 19 | Bicuspid aortic valve with mild stenosis.  Dilated ascending aorta. | 176 | 59 | 89 | Tachycardic with irregular heart rate |
| Patient 23 | M | 43 | Coarctation of the aorta.  Bicuspid aortic valve, moderate regurgitation and stenosis.  Subaortic stenosis.  S/P: Subclavian flap repair. Resection of subaortic ridge. | 174 | 68 | 67 |  |
| Patient 24 | M | 43 | Coarctation of the aorta. | 182 | 64 | 66 |  |
| Patient 25 | F | 32 | Functionally bicuspid aortic valve with moderate stenosis.  Mildly dilated ascending aorta | 158 | 70 | 85 |  |
| Patient 26 | F | 24 | Coarctation of the aorta.  S/P balloon dilatation. | 180 | 77 | 71 |  |
| Patient 27 | M | 26 | Aortic stenosis.  Bicuspid aortic valve with moderate stenosis and severe regurgitation.  Dilated aortic root and ascending aorta. | 180 | 80 | 66 |  |
| Patient 28 | M | 61 | Marfan syndrome.  Dilated aortic root.  S/P:Ascending aorta interposition graft. | 192 | 82 | 72 |  |
| Patient 29 | F | 41 | Functionally bicuspid aortic valve with severe stenosis and moderate regurgitation.  Moderate dilatation of the ascending aorta  Mild aortic root dilatation | 167 | 60 | 48 |  |
| Patient 30 | M | 41 | Mixed unicuspid aortic valve disease.  Aortic root aneurysm. | 173 | 86 | 69 |  |

Supplementary Table 2. Image quality scores analysis with respect to sharpness of the vessel (1 = non diagnostic, 4 = excellent) for the proposed iT2Prep-BOOST in comparison to the clinical T2Prep-bSSFP bright-blood images, indicating median (interquartile range). Scores for the three reviewers are shown, at the mid descending aorta (mDA), mid aortic arch (mAAr), mid ascending aorta (mAA) and aortic root (ARoot) levels

|  | T2Prep-bSSFP | iT2Prep-BOOST | p |
| --- | --- | --- | --- |
| **Reviewer 1** |  |  |  |
| ARoot | 3(2,3) | 4(4,4) | <0.05 |
| mAA | 3(2,3) | 4(4,4) | <0.05 |
| mAAr | 3(2,3) | 4(4,4) | <0.05 |
| mDA | 3(3,3) | 4(4,4) | <0.05 |
| **Reviewer 2** |  |  |  |
| ARoot | 4(4,4) | 4(4,4) | 0.50 |
| mAA | 4(3,4) | 4(4,4) | <0.05 |
| mAAr | 4(4,4) | 4(4,4) | 0.50 |
| mDA | 4(4,4) | 4(4,4) | 0.12 |
| **Reviewer 3** |  |  |  |
| ARoot | 4(3,4) | 4(4,4) | <0.05 |
| mAA | 4(3,4) | 4(4,4) | 0.43 |
| mAAr | 4(4,4) | 4(4,4) | 0.45 |
| mDA | 4(3,4) | 4(4,4) | 0.14 |

Supplementary Table 3. Image quality scores analysis with respect to presence of artefacts (1 = non diagnostic, 4 = excellent) for the proposed iT2Prep-BOOST in comparison to the clinical T2Prep-bSSFP bright-blood images, indicating median (interquartile range). Scores for the three reviewers are shown, at the mid descending aorta (mDA), mid aortic arch (mAAr), mid ascending aorta (mAA) and aortic root (ARoot) levels

|  | T2Prep-bSSFP | iT2Prep-BOOST | p |
| --- | --- | --- | --- |
| **Reviewer 1** |  |  |  |
| ARoot | 3(2,3) | 4(4,4) | <0.05 |
| mAA | 3(2,3) | 4(4,4) | <0.05 |
| mAAr | 3(2,3) | 4(4,4) | <0.05 |
| mDA | 3(3,3) | 4(4,4) | <0.05 |
| **Reviewer 2** |  |  |  |
| ARoot | 4(3,4) | 4(4,4) | <0.05 |
| mAA | 3(3,4) | 4(4,4) | <0.05 |
| mAAr | 4(3,4) | 4(4,4) | <0.05 |
| mDA | 4(3,4) | 4(4,4) | <0.05 |
| **Reviewer 3** |  |  |  |
| ARoot | 3(3,4) | 4(4,4) | <0.05 |
| mAA | 4(3,4) | 4(4,4) | <0.05 |
| mAAr | 4(3,4) | 4(4,4) | <0.05 |
| mDA | 4(4,4) | 4(4,4) | 0.37 |

Supplementary Table 4. Upper and lower limits of agreement for co-axial aortic diameter measurements at the aortic root (ARoot), mid ascending aorta (mAA) and mid descending aorta (mDA), with iT2Prep-BOOST vs T2Prep-bSSFP approach for two reviewers.

|  | Upper limit of agreement (+1.96 SD) | Lower limit of agreement (-1.96 SD) |
| --- | --- | --- |
| **Reviewer 2** |  |  |
| ARoot | 2.4mm | -2.2mm |
| mAA | 2.3mm | -2.0mm |
| mDA | 1.9mm | -2.0mm |
| **Reviewer 3** |  |  |
| ARoot | 1.9mm | -1.7mm |
| mAA | 1.3mm | -1.5mm |
| mDA | 1.8mm | -1.4mm |

Supplementary Table 5. Upper and lower limits of agreement for intra-rater co-axial aortic diameter measurements at the aortic root (ARoot), mid ascending aorta (mAA) and mid descending aorta (mDA) with the iT2Prep-BOOST approach.

|  | Upper limit of agreement (+1.96 SD) | Lower limit of agreement (-1.96 SD) |
| --- | --- | --- |
| ARoot | 1.5mm | -2.1mm |
| mAA | 1.8mm | -2.0mm |
| mDA | 1.8mm | -2.1mm |

Supplementary Table 6. Intraclass Correlation Coefficient (ICC) for inter-reviewer (Reviewer 2 and 3) and intra-reviewer (Reviewer 2) agreement and 95% Confidence Interval (CI) for aortic dimension measurements performed on bright-blood iT2Prep-BOOST images at the aortic root (ARoot), mid ascending aorta (mAA) and mid descending aorta (mDA).

|  | Inter-observer ICC  (95% CI) | Intra-observer ICC  (95% CI) |
| --- | --- | --- |
| ARoot | 0.96 (0.93, 0.98) | 0.96 (0.93, 0.98) |
| mAA | 0.98 (0.97, 0.99) | 0.99 (0.98, 0.99) |
| mDA | 0.94 (0.89, 0.94) | 0.96 (0.93, 0.98) |

**Supplementary Figures**


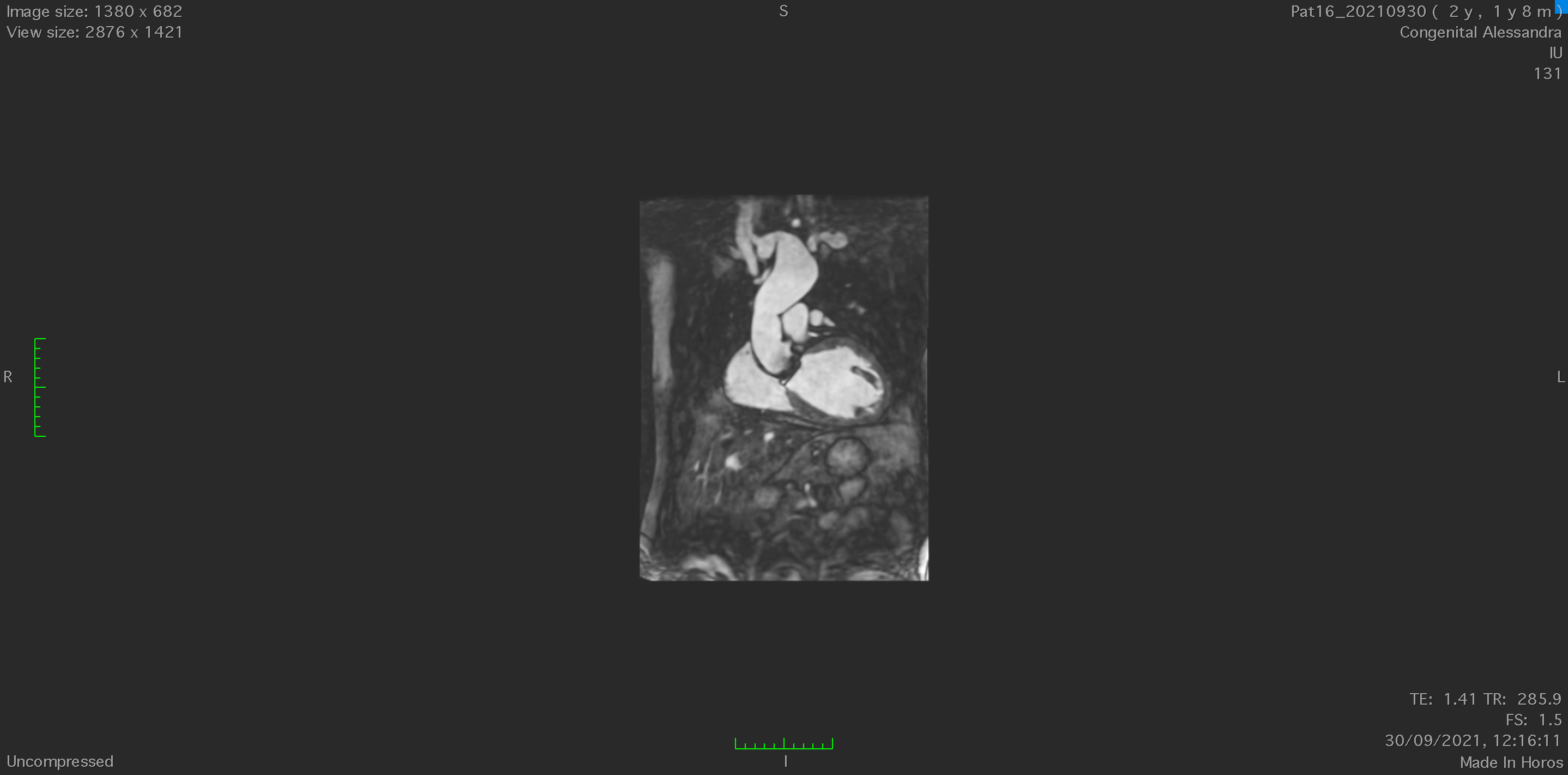

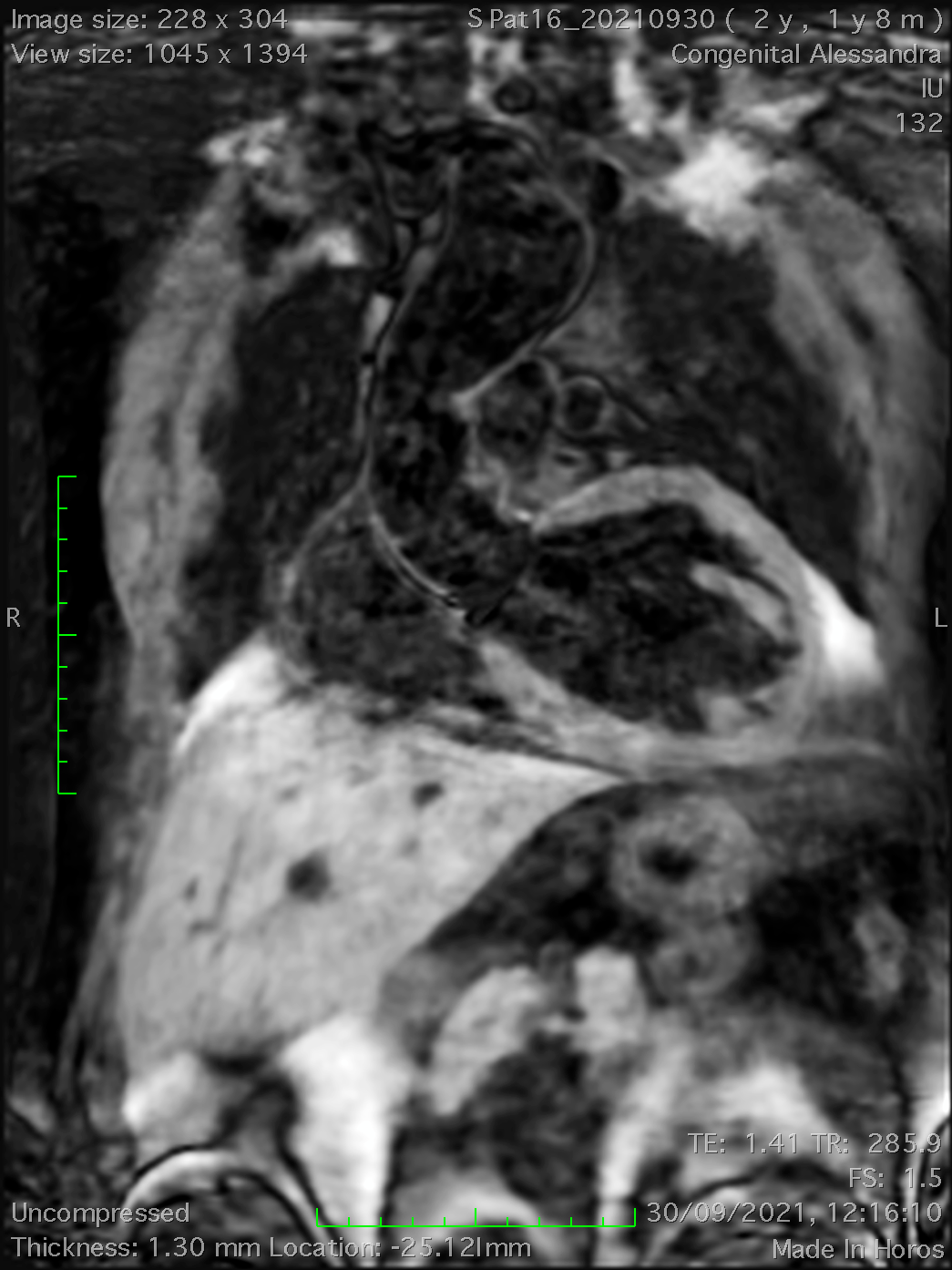


Supplementary Figure 1. Coronal view of the left ventricular outflow tract and the aorta in a 52-year-old patient with Marfan’s syndrome and mechanical aortic valve in situ. The mechanical valve can be visualized in the bright-blood iT2prep-BOOST dataset with minimal artefact in the surrounding tissues. The black-blood iT2prep-BOOST nulls the signal from the mechanical valve.
